# Membrane trafficking in breast cancer progression: protein kinase D comes into play

**DOI:** 10.3389/fcell.2023.1173387

**Published:** 2023-05-24

**Authors:** Elena Gutiérrez-Galindo, Zeynep Hazal Yilmaz, Angelika Hausser

**Affiliations:** ^1^ Institute of Cell Biology and Immunology, University of Stuttgart, Stuttgart, Germany; ^2^ Stuttgart Research Center Systems Biology, University of Stuttgart, Stuttgart, Germany

**Keywords:** PKD, breast cancer, membrane trafficking, epithelial-to-mesenchymal transition, trans-Golgi network

## Abstract

Protein kinase D (PKD) is a serine/threonine kinase family that controls important cellular functions, most notably playing a key role in the secretory pathway at the trans-Golgi network. Aberrant expression of PKD isoforms has been found mainly in breast cancer, where it promotes various cellular processes such as growth, invasion, survival and stem cell maintenance. In this review, we discuss the isoform-specific functions of PKD in breast cancer progression, with a particular focus on how the PKD controlled cellular processes might be linked to deregulated membrane trafficking and secretion. We further highlight the challenges of a therapeutic approach targeting PKD to prevent breast cancer progression.

## 1 Introduction

Intracellular membrane trafficking is the process by which proteins and macromolecules are distributed throughout the cell and released to or internalized from the extracellular space. It can be classified into two major pathways, the biosynthetic exocytic trafficking pathway and the endocytic trafficking pathway. Exocytosis refers to the transport of cargo to the plasma membrane or out of the cell. In this process, newly synthesized proteins, lipids or carbohydrates are transported from the endoplasmic reticulum (ER) via the Golgi complex to the cell membrane or into the extracellular space. The integrity of these organelles defines the structure and sub-compartmentalisation for proper trafficking. In addition, some cargo molecules are also transported out of the cell via unconventional exocytosis pathways that are independent of the ER and/or the Golgi complex, such as direct translocation of soluble components across the plasma membrane, release via secretory endosomes and lysosomes, and extracellular vesicles (EVs). On the other hand, endocytosis describes the internalization of cargo from the plasma membrane into the cell. This serves to absorb nutrients and pass cargo for recycling or degradation by lysosomes. However, some endosomal proteins escape from this pathway and are delivered to the trans-Golgi network (TGN) either by the early endosome/recycling endosome or by the late endosome (Herrmann and Spang, 2015; Rabouille, 2017; Tu et al., 2020; Yarwood et al., 2020). Finally, to maintain homeostasis, cells degrade dysfunctional internal components such as damaged organelles and protein aggregates by autophagy, whereas proteins that do not fold properly are subject to ER-associated degradation (ERAD), in which they are ubiquitinylated, extracted from the ER, and degraded by the proteasome (Moon et al., 2018; Benyair et al., 2022).

**FIGURE 1:**
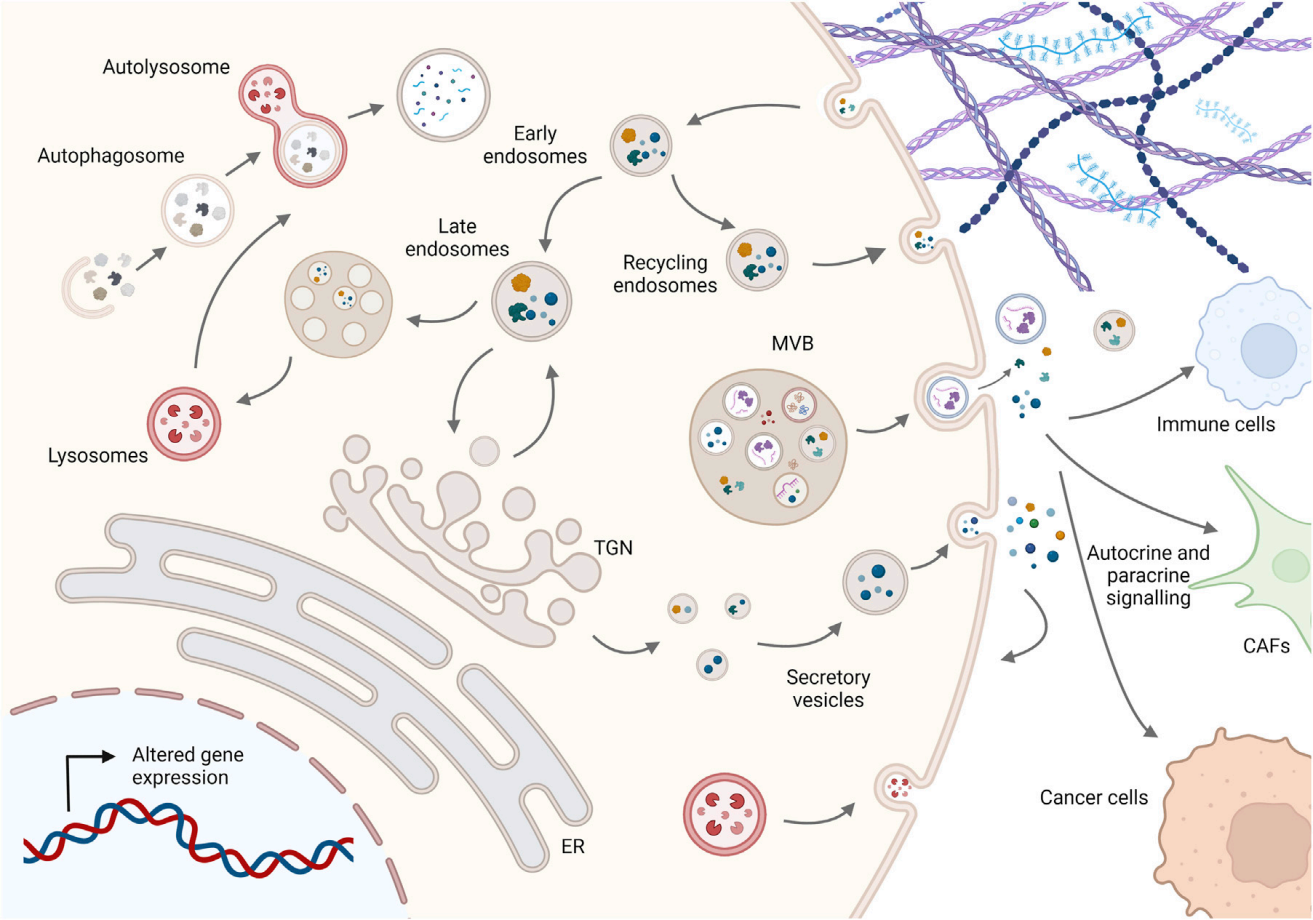
Schematic representation of membrane trafficking pathways in cancer cells. Molecules are secreted either by the conventional pathway involving the ER, Golgi complex, and secretory vesicles or by diverse unconventional pathways such as multivesicular bodies (MVBs) secreting EVs or lysosomal secretion. On the other hand, molecules are taken up by endocytosis and can be recycled to the plasma membrane or degraded via the endolysosomal system, while dysfunctional internal components are degraded by fusion of autophagosomes with lysosomes to maintain cellular homeostasis. Imbalance in this dynamic membrane trafficking promotes cancer progression by altering the presentation and degradation of key membrane proteins, cellular proteostasis, autocrine and paracrine signaling to other cells such as cancer-associated fibroblasts (CAFs) and immune cells, and ECM composition and structure. For simplicity, not all membrane trafficking pathways are shown; see text for more details. Created in BioRender.

Membrane trafficking is coordinated through a complex network of signaling pathways and serves to maintain cellular homeostasis. Consequently, dysregulation of proteins and signaling pathways through mutations, chromosomal rearrangements, aberrant gene expression, or epigenetic changes promotes aberrant expression of transmembrane proteins, cargo sorting and transport and is thus a key factor in the development of diseases such as neurodegeneration and immune disorders (Wang et al., 2014; Taguchi and Mukai, 2019). In recent years, dysregulated membrane trafficking has also emerged as a driver of cancer progression. Alterations in the presentation and degradation of key membrane proteins and an imbalance in dynamic vesicle trafficking processes are known to be critical for tumor progression (Cho et al., 2018; Drizyte-Miller et al., 2020; Yousaf & Ali, 2020), as they contribute to several features of cancer, including hyperproliferation, evasion of growth suppression, loss of cell polarity, activation of cell motility, invasion and metastasis, shaping of the tumor microenvironment (TME), and resistance to drug-induced cell death [Figure 1, reviewed in (Tejeda-Muñoz et al., 2022)]. For example, increased expression of the small GTPase Rab2A was found in breast cancer tissue compared with adjacent normal breast tissue and was significantly associated with poor prognostic markers manifesting Rab2A as an independent predictor of disease recurrence in breast cancer patients. At the molecular level, Rab2A regulates endocytic recycling of MT1-MMP, transport of E-cadherin to the Golgi complex and promotes Erk signalling in breast cancer cells (Kajiho et al., 2016), enabling their mesenchymal invasion and increasing the population of breast cancer stem cells (CSCs) (Luo et al., 2015; Kajiho et al., 2016; 2018). Another example of how deregulated transport can promote cancer progression is the Golgi-localized phosphatidylinositol 4-phosphate effector protein GOLPH3, which is responsible for retrograde transport of glycosyltransferase, thereby controlling the lysosomal degradation of these enzymes. Overexpression of GOLPH3 has been demonstrated in several human solid cancers, including breast cancer, glioblastoma, gastric cancer, and colorectal cancer, and is associated with cancer progression (Sechi et al., 2020). Indeed, the aberrant overexpression of GOLPH3 increases glycosyltransferase retention in the Golgi, thereby promoting biosynthesis of glycosphingolipids leading to enhanced signaling of receptor tyrosine kinases and concomitantly cell growth and survival (Chen et al., 2012; Rizzo et al., 2021; Sechi et al., 2020). Additionally, with increasing aberrant secretion, EVs released by cancer cells containing pro-oncogenic molecules can control tumor growth, metastasis, and the TME through paracrine signaling (Green et al., 2015; Becker et al., 2016; Ciardiello et al., 2016). This aberrant EV secretion can be used as a biomarker for breast cancer screening of patients (Chen et al., 2017). Finally, altered extracellular matrix (ECM) secretion and aberrant ECM formation at the TME causes epithelial to mesenchymal transition (EMT), promotes cancer-stem-like properties in the surrounding cells (Liu et al., 2019), and is linked with drug resistance (Dinca et al., 2021; Papanicolaou et al., 2022).

Among the key proteins regulating membrane trafficking at the level of the TGN are the three members of the protein kinase D (PKD) family of serine/threonine kinases, PKD1, PKD2 and PKD3. In this review, we will discuss how PKD regulates membrane trafficking along endocytic and exocytic pathways and how aberrant PKD activity in particular may contribute to cancer progression by potentially altering membrane trafficking. We will also address the technical challenges associated with linking kinase localization to function and ultimately identifying isoform-specific functions.

## 2 PKD activation and function in membrane trafficking

**FIGURE 2:**
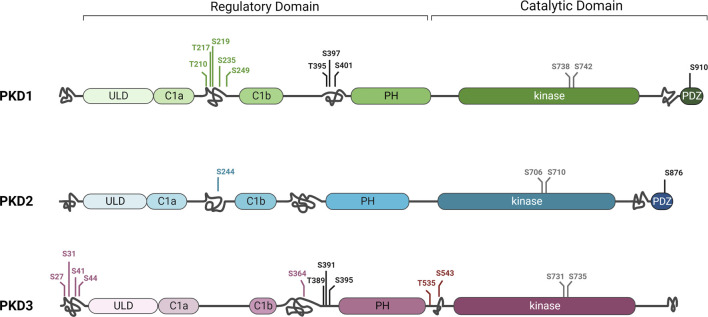
Differences and similarities in protein kinase D family structure. PKD1, 2, and 3 share highly conserved sequences in the C1A and C1B, ULD, PH, and kinase domains. However, they also have some differences in structure, such as the PDZ domain and a C-terminal autophosphorylation site that are present only in PKD1 and PKD2. The three isoforms also differ in putative disordered domains (intertwined lines) in which unique phosphorylation sites (green, blue, or purple) or phosphorylation sites present in only two of the isoforms (black) were detected in different regions. Full activity of the kinase depends on phosphorylation of two conserved serines of the activation loop in the kinase domain (grey). For simplicity, only some phosphorylation sites are shown in this figure. Additional phosphorylations as well as other posttranslational modifications were detected (Hornbeck et al., 2015). See text for details. Created in BioRender.

The three PKD isoforms are structurally very similar: the N-terminal regulatory domain contains two C1 domains, C1a and C1b, that bind to the lipid second messenger diacylglycerol (DAG) (Baron and Malhotra, 2002), a ubiquitin-like domain (ULD) that promotes homo- and heterodimerisation of the kinases (Aicart-Ramos et al., 2016; Elsner et al., 2019), and an autoinhibitory pleckstrin homology (PH) domain (Iglesias and Rozengurt, 1998). The highly conserved kinase domain with the two activation loop serines is located at the carboxy terminus. However, there are also some differences: PKD1 and PKD2 contain an apolar and proline-rich region, respectively, in the amino-terminal domain, which is absent in PKD3 (Sánchez-Ruiloba et al., 2006). Similarly, PKD1 and PKD2, but not PKD3, contain binding motifs for postsynaptic density protein-95/disc-large tumor suppressor protein/zonula occludens-1 (PDZ) at their carboxyl terminals, which can direct the kinases to different subcellular scaffolds through interactions with PDZ domain-containing proteins (Sánchez-Ruiloba et al., 2006; Kunkel et al., 2009). The structural similarities of PKD1 and PKD2 can be explained by phylogenetic analyses showing that PKD1 and PKD2 are descended from a common ancestor, while PKD3 arose earlier (Reinhardt et al., 2020). All three isoforms are also predicted to have intrinsically disordered domains at different positions that, interestingly, contain specific phosphorylation sites unique to each isoform (Figure 2). For example, in PKD2, CK1-mediated phosphorylation of S244, which is not found in PKD1 and 3, caused translocation to the nucleus (Von Blume et al., 2007). Additionally, for PKD1, several unique phosphorylation sites have been discovered between C1a and C1b, and in PKD3, at least four unique phosphorylations have been identified to date in the C-terminus, as well as two others flanked by the PH and kinase domains (Daub et al., 2008; Dephoure et al., 2008; Franz-Wachtel et al., 2012; Klammer et al., 2012; Schweppe et al., 2013; Mertins et al., 2014) (Figure 2). In most cases, these phosphorylation sites have been detected by mass spectrometry analysis and no specific function has yet been attributed to them, so it is not known whether they actually affect PKD localization and/or activity. Of note, posttranslational modifications occurring in intrinsic disordered regions can increase the functional states in which a protein can exist in the cell (Collins et al., 2008) and may thus provide an explanation for non-redundant functions of the PKD isoforms (Huck et al., 2012; 2013; Borges et al., 2015). Further studies are therefore needed to understand whether and how these differences in phosphorylation patterns within disordered domains might affect the function of the three PKD isoforms.

Binding of PKD to DAG promotes phosphorylation of the two activation loop serines, making the kinase fully active and enabling phosphorylation of its downstream substrates. Consequently, PKD activation is downstream of signalling events that trigger the production of DAG, e.g., G-protein-coupled receptor (GPCR) and receptor tyrosine kinase-induced phospholipase-C (PLC) signalling or RhoGTPase signaling. Given the variety of stimuli that lead to PKD activation, it is not surprising that the kinase family is involved in such diverse cellular functions as membrane trafficking, actin remodelling and regulation of gene expression (Fu and Rubin, 2011). It has long been assumed that members of the novel protein kinase C (nPKC) family, which are also activated by binding to DAG, in turn phosphorylate the activation loop serines thereby activating PKD (Díaz-Añel and Malhotra, 2005; Jacamo et al., 2008). However, recent research has shed new light on the activation mechanism of PKD isoforms and questioned a role for nPKCs. Elsner and co-workers reported that PKD dimerisation via the ULD is a prerequisite for autophosphorylation of the activation loop (Elsner et al., 2019). This is consistent with a number of studies showing that dimerisation of PKD is required for its cellular function (Bossard et al., 2007; Döppler et al., 2014; Aicart-Ramos et al., 2016). Biochemical experiments with recombinant proteins have clearly shown that PKD isoforms can form hetero- and homodimers (Bossard et al., 2007; Aicart-Ramos et al., 2016; Elsner et al., 2019), but they also have limitations with regard to conclusions on the dimerisation of endogenous PKD isoforms in intact cells. It is therefore not yet clear whether only the expression pattern and level of PKD isoforms determines which dimers form, or whether other factors, such as structural differences, or interaction with other proteins, also play a role. Thus, it is conceivable that depending on the dimer formed, the PKD localization, and consequently the function, would be different. For example, while PKD1 has tumor promoting functions in pancreatic cancer, it shows tumor suppressive functions in breast cancer (Eiseler et al., 2009a; Harikumar et al., 2010; Ochi et al., 2011; Borges et al., 2013). Do the protein expression levels of PKD2 and PKD3 in these tumor entities dictate which homodimer and/or heterodimer forms? To shed light on this, CRISPR/Cas technology can now be used to tag the endogenous PKD isoforms with various fluorescent marker proteins and then use high-resolution microscopy to track their subcellular localization and dimerization spatially and temporally in different cell types.

While initially speculated that dimer formation occurs upon binding to DAG (Elsner et al., 2019), a new study by Reinhard and coworkers now shows that cytosolic PKD is a constitutive dimer in which its kinase domains are in a trans-autoinhibitory face-to-face dimer (Reinhardt et al., 2023). Binding to DAG leads to conformational changes that abolish inhibitory dimerisation of the kinase domains and result in cis-autophosphorylation of the activation loop. The phosphorylation of the activation loop promotes then both the trans-phosphorylation of substrates and the prevention of the reassembly of the kinase domains (Elsner et al., 2019; Reinhardt et al., 2023). In line with this model, active PKD is mainly found on membranes, while cytosolic PKD remains inactive (Hausser et al., 2002). Considering the multiple functions of PKD and the increasing importance of deregulation of kinase activity in disease development, the concept of independent auto-activation is logical. However, phosphorylation by kinases at other sites might still modulate protein interactions of PKD family members thereby fine-tuning activity (Hausser et al., 1999; Eisenberg-Lerner & Kimchi, 2007; Durand et al., 2016). Since DAG production occurs also in organelles at which, at least to date, PKD has not been found such as the ER (Eichmann and Lass, 2015), the question arises whether other components determine the specific localization of PKD. Indeed, the small GTPase ARF1 has been reported to bind to all three PKD isoforms *in vitro* and this interaction seems to be required to target the kinases to the TGN (Varma Pusapati et al., 2010). ARF1 binds to the C1b domain of PKD, which, however, also binds DAG (Baron & Malhotra, 2002). Therefore, no conclusion can be drawn as to which of the two interactions is crucial for TGN localization of PKD. In addition, ARF1 is not found at mitochondria or the plasma membrane, leaving room for speculation that other proteins exist that act as spatial cues for PKD localization at these organelles. Through structural analysis of the C1a domain, Leonard and colleagues identified a basic pocket near the DAG-binding cleft that could potentially bind acidic phospholipids thereby supporting organelle-specific DAG binding (Reinhardt et al., 2020). Although there is no experimental evidence for this to date, it is conceivable that acidic phospholipids such as phosphatidic acid, which plays an important role in TGN membrane fission (Pagliuso et al., 2016), may further support PKD membrane binding. Nevertheless, binding to membrane-embedded DAG constitutes as major factor responsible for PKD activation and the presence and amount of DAG in intracellular membranes thus determines the activity level of the PKD dimer.

Because different signaling pathways can trigger the production of DAG at distinct organelles, different localizations of PKD family members have been found, such as mitochondria, plasma membrane, and TGN, often depending on cell type and stimulus (Prestle et al., 1996; Liljedahl et al., 2001; Cowell et al., 2009; Chang et al., 2017). The signaling pathways and the cellular outcome triggered by PKD activity are then determined by the substrates available at the corresponding organelle. For example, PKD1 and PKD2 are recruited to the outer mitochondrial membrane under oxidative stress conditions by phospholipase D (PLD)-mediated DAG production (Cowell et al., 2009; Cobbaut et al., 2017). Both isoforms are further modulated in their activity by tyrosine phosphorylation and contribute to the detoxification and survival of the cell by activating various downstream signalling pathways [detailed review in (Cobbaut and Lint, 2018)]. PKD1 was also reported to be involved in endosomal trafficking of plasma membrane-localized receptors such as integrins and AMPA receptors, presumably through phosphorylation of the Rab5 effector protein rabaptin-5 (Woods et al., 2004; Christoforides et al., 2012; Oueslati Morales et al., 2021). Consistent with this, ectopically expressed, kinase-inactive PKD1 was detected on endocytic vesicles that are downstream of a Rab4-dependent transport step (Woods et al., 2004).

One of the first described and to date best-understood functions of PKD is the regulation of constitutive secretory transport at the level of the TGN. All three isoforms localize to the TGN when ectopically expressed in HeLa cells (Hausser et al., 2002). At the TGN, PKD assists in the fission of vesicles carrying specific cargo molecules destined for the plasma membrane (Liljedahl et al., 2001; Yeaman et al., 2004). This is achieved through a complex network of the activity of several downstream, TGN-localized substrates such as the lipid kinase PI4KIIIβ and the lipid transfer proteins CERT and OSBP, which modulate the lipid content of the TGN membrane to enable vesicle formation and fission and are interconnected by positive and negative feedback loops [extensively reviewed in (Wakana and Campelo, 2021)]. This function in constitutive secretion is also consistent with the binding to and activation of PKD by DAG as the conical shape of DAG in the outer leaflet provides negative curvature of the membrane, which presumably facilitates membrane cleavage (Szule et al., 2002). Furthermore, PKD activity is triggered during cargo trafficking, implying that cargo initiates pathways to generate DAG (Di Martino et al., 2020). Recently, it has also been reported that PKD is involved in the formation of tubular-vesicular carriers through the phosphorylation of PARP12, a mono-ADP-ribosyltransferase localized at the TGN in MCF-7 cells. Phosphorylated PARP12 catalyzes mono-ADP-ribosylation of Golgin-97, which in turn promotes the formation of carriers that transport E-cadherin to adherens junctions (Grimaldi et al., 2022), adding a further layer of complexity to the mechanism by which PKD controls membrane fission. Accordingly, the transport of various cargo proteins, from soluble factors such as PAUF and lysozyme C to plasma membrane-localised transmembrane proteins such as E-cadherin, CD4 and β1 integrin, has been linked to PKD activity (Wakana et al., 2012; Wakana & Campelo, 2021; Grimaldi et al., 2022), revealing the kinase family as a master regulator of TGN function. However, as mentioned above, it is not yet clear whether all three PKD isoforms contribute similarly or exert isoform-specific functions, because several studies have used overexpression of kinase-dead PKD constructs that block secretion in a dominant-negative manner by dimerizing with endogenous PKD proteins (Elsner et al., 2019). Considering that HeLa cells, which have often been used for studies on PKD function at the TGN, do not express PKD1 (Bossard et al., 2007), regulation of secretion in these cells could be attributed to PKD2/PKD3 homo- or heterodimers. Indeed, Bossard and colleagues showed that loss of either isoform blocked secretion of the artificial cargo protein ssHRP in HeLa cells (Bossard et al., 2007). This suggests that active PKD2/3 heterodimers are present at the TGN and regulate the fission of transport vesicles. Intriguingly, depletion of PKD2 in HEK293T cells was sufficient to abrogate ssHRP secretion (Weeber et al., 2019), although PKD1 and PKD3 are expressed in these cells (Hausser et al., 2005). Finally, depletion of PKD2 and PKD1 alone or in combination abrogated Golgin-97 MARylation in MCF-7 cells that do not express PKD3, and consequently Golgin-97-controlled E-cadherin transport (Grimaldi et al., 2022). Taken together, these data may indicate that PKD2/PKD3 and PKD1/PKD2 heterodimers regulate the export of cargo at the TGN. However, whether the downstream substrate and transported cargo is dependent on the dimerization partner is an exciting question that still awaits investigation.

## 3 PKD function in breast cancer—a role for membrane trafficking?

In recent years, aberrant expression and activity of PKD isoforms has been detected in various cancers, such as prostate, breast, skin or pancreatic cancer (Roy et al., 2017) making the kinase family a potential target for cancer therapy. In breast cancer, the three PKD isoforms exhibit distinct expression patterns and regulate different tumor suppressive and oncogenic processes. In highly invasive breast cancer, loss of PKD1 appears to promote invasion and metastasis, whereas PKD2 and upregulated PKD3 positively influence proliferation, chemoresistance and metastasis (Borges and Storz, 2013). The signaling pathways regulated by PKD in this context were associated with transcriptional regulation of gene expression, EMT or dynamic regulation of the actin cytoskeleton [reviewed in (Fu & Rubin, 2011; Olayioye et al., 2013)] amongst others, whereas the role of PKD-dependent membrane trafficking was largely neglected. However, some PKD activators and substrates that play an important role in membrane trafficking have also been attributed tumor-promoting properties, particularly in breast cancer, implying that PDK-regulated membrane trafficking may contribute to breast cancer progression. For example, PI4KIIIβ, which generates phosphatidylinositol-4-phosphate (PI4P) and vesicle carriers at the TGN and whose activity is stabilized by PKD-mediated phosphorylation (Hausser et al., 2005; 2006), is overexpressed in breast cancer tumors (Morrow et al., 2014). Importantly, loss of PI4KIIIβ expression or inhibition of its catalytic activity can result in decreased cell proliferation and reduced cell survival, suggesting that the lipid kinase has a pro-oncogenic function (Chu et al., 2010). Additionally, the protein expression of the RhoGEF GEF-H1, which is upstream of PKD activity at the TGN (Eisler et al., 2018), is upregulated in human breast tumors and surrounding stromal cells (Cheng et al., 2008).

Below, we provide an overview of the known functions attributed to PKD in breast cancer and the similarities with other cancers that may be useful in understanding the complex role of PKD. We also refer to other reviews addressing this topic in more detail (Durand et al., 2015; Roy et al., 2017). We further critically discuss the evidence for the involvement of PKD-regulated membrane trafficking in breast cancer and highlight the challenges of a therapeutic approach targeting PKD to prevent breast cancer progression.

### 3.1 A PKD isoform switch occurs in breast cancer

Breast cancer is the most common cancer among all other cancers with an incidence of 11.7% (Sung et al., 2021). Breast cancer is a genetically and clinically heterogeneous disease with multiple subtypes. The most common and widely accepted classification of breast cancer is from an immunohistochemical perspective and is based on the expression of the following hormone receptors: estrogen (ER), progesterone (PR), and human epidermal growth factor 2 (HER2). Accordingly, the following subtypes of breast cancer are generally recognized: luminal A, (ER/PR+, HER2−, Ki67 low), luminal B (ER+, HER2−, Ki67 high or PR- and ER+, HER2+), HER2 overexpressed (ER/PR−, HER2+), and basal-like triple negative breast cancer (TNBC) (ER−, PR−, HER2−) (Yin et al., 2020). Because TNBC tends to grow and spread more rapidly than other breast cancer subtypes, it is considered a particularly aggressive form of breast cancer. Accordingly, breast cancer patients tend to develop metastases and have a poor prognosis. Moreover, targeted therapies are limited due to the loss of ER, PR and HER2 receptors, and non-selective chemotherapy is still the main treatment option (Vagia et al., 2020).

**FIGURE 3:**
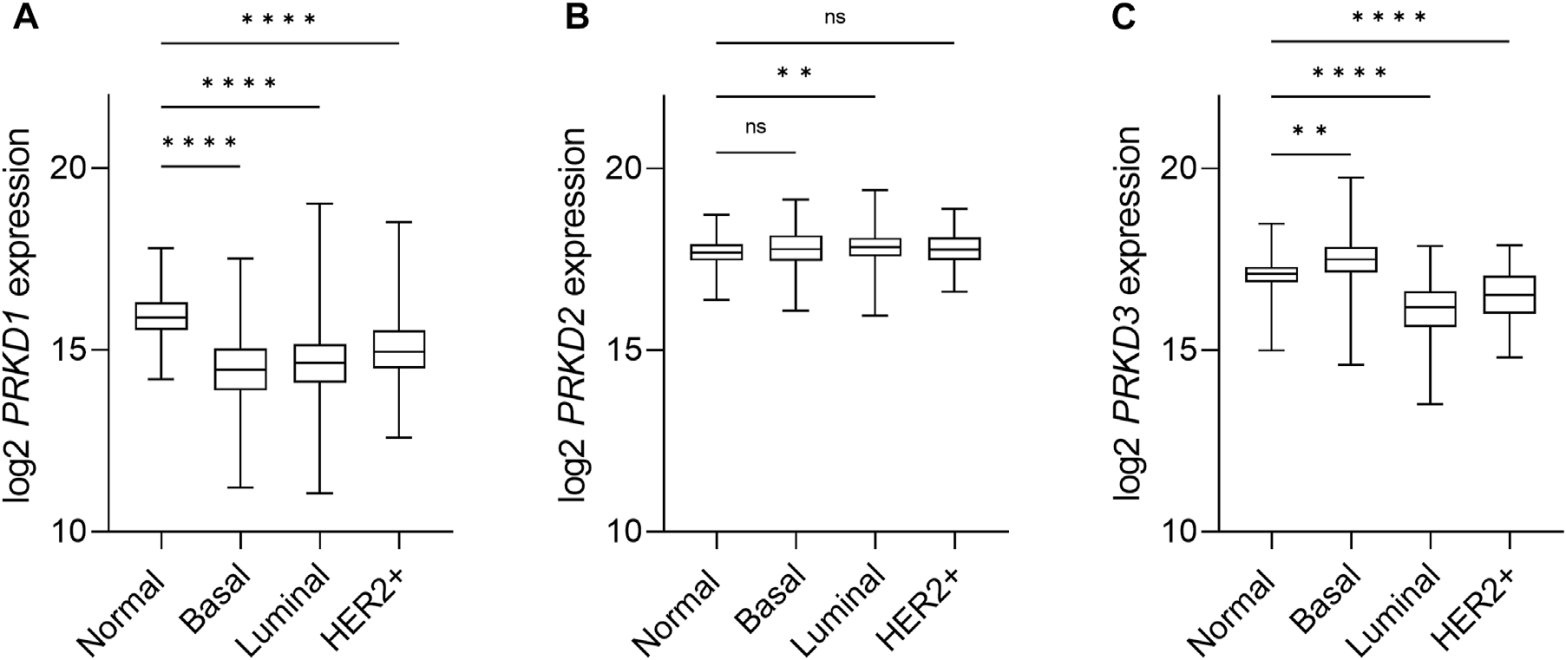
Expression of PKD isoforms in breast cancer subtypes. Box-and-whisker plots representing the relative mRNA expression levels of the *PRKD1* **(A)**, *PRKD2* **(B)** and *PRKD3* **(C)** genes, encoding for the PKD1, PKD2 and PKD3, respectively, distributed across the PAM50 breast cancer molecular subtypes (Berger et al., 2018). Target gene expression data was retrieved from the TCGA-BRCA (The Cancer Genome Atlas Breast Cancer) RNAseq dataset and the sample cohort was divided within subtypes based on the available PAM50 annotation within the dataset, as follows: Normal/Normal-like (n = 153), Basal (n = 194), Luminal (n = 774), Her2+ (n = 82). Luminal subtypes A (n = 567) and B (n = 207), as well as normal tissue samples (n = 113) and normal-like (n = 40) samples, were combined for the sake of clarity. n: number of samples in the subgroup. Statistical analysis was done using the Kruskal Wallis test in GraphPad Prism 9. ****p < 0.0001, **p = 0.0061, ns, not significant.

An isoform-specific expression pattern was described in breast cancer patients that correlates with aggressiveness. While PKD1 was the dominant isoform in normal breast tissue, samples of invasive ductal carcinoma showed a switch towards PKD3 expression, and a weaker expression of PKD2 (Eiseler et al., 2009a; Borges et al., 2015). This first observation in patient samples was followed by the study of the isoform-specific transcripts and protein expression of the PKD family in a panel of breast cancer cell lines in this and other studies, with an inverse correlation of PKD1 and PKD3 expression in TNBC cell lines in comparison to non-TNBC lines. The observed changes in PKD2 expression are less consistent, as in general PKD2 levels remain more stable (Chen et al., 2011; Hao et al., 2013; Huck et al., 2013; Borges et al., 2015). We conducted an analysis of PKD mRNA expression levels in breast cancer using the TCGA database that revealed a decrease in PKD1 expression and an increase in PKD3 expression, whereas PKD2 levels are unchanged in basal-like breast cancer compared with normal tissue (Figures 3A–C). Although it is not clear whether the increased mRNA level translates into an enhanced protein level, this supports the idea that PKD1 is a potential tumor suppressor, whereas PKD3 has oncogenic functions in breast cancer.

A similar expression pattern is also observed in other tumor types: in colorectal cancer (CRC), PKD1 is expressed only in normal colon cells, PKD2 is the dominant isoform at the transcriptional level in tumor samples, and both PKD2 and PKD3 are highly expressed in CRC cell lines (Wei et al., 2014). Primary gastric tumors and gastric cancer cell lines also have low levels of PKD1 (Kim et al., 2008), and PKD1 is downregulated in androgen-independent prostate cancers (Jaggi et al., 2003), as well as in non-small cell lung cancer (NSCLC) (Ni et al., 2015). In pancreatic cancer, however, PKD1 appears to be the dominant isoform in highly invasive cell lines and tumor samples (Harikumar et al., 2010; Ochi et al., 2011). These differences in expression depending on tissue or cell type also support the idea that distinct dimers exist which might exert isoform-specific functions.

### 3.2 PKD1 as a potential tumor suppressor in breast cancer

In 2009, Eiseler and colleagues identified that the *PRKD1* promoter was aberrantly methylated in highly invasive breast cancer cell lines, leading to a decrease in the protein expression (Eiseler et al., 2009a). This observation was later validated in breast cancer patient samples, where methylation levels correlated with invasiveness and metastasis. Furthermore, pharmacologic reversion of *PRKD1* methylation with the DNA methyltransferase inhibitor decitabine restored PKD1 expression and reduced tumor invasion and metastasis to lungs in an animal model (Borges et al., 2013). The inactivation of PKD1 expression via *PRKD1* methylation was also found in primary gastric tumors and gastric cancer cell lines, and significantly correlated with age (Kim et al., 2008). Consistent with these results, ectopic expression of PKD1 in the highly invasive TNBC cell line MDA-MB-231 resulted in a decrease in invasiveness, whereas silencing of PKD1 in the non-TNBC cell line MCF-7 in 2D and 3D had the opposite effect, suggesting that loss of PKD1 is a key marker of aggressiveness (Eiseler et al., 2009a). Regarding cell motility, different studies have shown a negative regulation of cell migration by PKD1. In the mouse-myoblast cell line pmi28, PKD1 negatively regulated cell migration by controlling dynamic changes in the actin cytoskeleton at the leading edge through direct binding to F-actin (Eiseler et al., 2007). In breast cancer cells, depletion of PKD1 stimulated migration in MCF-7 cells; however, the effect was higher when PKD2 was silenced (Peterburs et al., 2009). This feature of PKD has been widely studied and linked to actin remodelling at the leading edge of motile cells via different substrates, such as the phosphatase slingshot 1 like (SSH1L), which activates the actin depolymerizing factor cofilin and the nucleation promoting factor cortactin (Eiseler et al., 2009b; Peterburs et al., 2009). However, both actin-modulating factors also play a role in vesicle fission at the level of the TGN. Weeber and colleagues have shown that phosphorylation of cortactin mediated by PKD2 (and presumably PKD3) is required to allow vesicle formation until a fission-competent state is reached (Weeber et al., 2019). In addition, active ADF/Cofilin recruits F-actin to TGN membranes to assist in sorting cargo into nascent carriers (von Blume et al., 2009). Although it is not yet clear whether this occurs in a PKD-dependent manner, it supports the notion that the migration phenotype observed in PKD-depleted cells may also result, at least in part, from impaired cargo sorting and membrane fission required to support transport carrier biogenesis at the TGN.

PKD1 not only controls dynamic actin turnover and cell migration, but is also involved in maintaining epithelial phenotype and preventing EMT in various cancer cells. For example, direct interaction and phosphorylation of E-cadherin by PKD1 in adherent junctional sites resulted in increased cell aggregation and decreased cell motility in non-invasive LNCaP prostate cancer cells (Du et al., 2009). In another study using prostate cancer epithelial cells, PKD1 was shown to inhibit EMT by phosphorylating the transcription factor SNAI1, which promotes the repression of the adhesion molecule E-cadherin to regulate EMT, directly at Ser11, leading to its binding to 14-3-3 proteins and subsequent nuclear export of SNAI1. This resulted in a positive regulation of E-cadherin mRNA levels and stabilization of cell-cell contacts (Du et al., 2010). In line with this, analysis of two DNA microarray datasets with prostate cancer patients correlated PKD1 expression with higher E-cadherin expression levels and lower metastasis rates (Du et al., 2010). Besides, PKD-dependent phosphorylation of SNAI1 at Ser11 was also important for its interaction with the E3 ligase FBXO11, which ultimately led to SNAI1 ubiquitination and degradation. Furthermore, SNAI1 overexpression induced EMT and spontaneous lung metastases in mice, which was abolished upon parallel expression of FBXO11. Strikingly, SNAI1 protein expression levels correlated with lymph node invasion while a negative correlation of active PKD1 and FBXO11 with SNAI1 was observed in clinical breast cancer samples (Zheng et al., 2014). However, it has also been reported that phosphorylation of SNAI1 is not a prerequisite for its degradation (Jin et al., 2015), so the described mechanism may be unique to PKD1-positive breast cancer. Nevertheless, these data further support the role of PKD1 as a tumor suppressor in breast cancer. In line with this, Lu and collegues concluded that inhibition of bromodomain-containing protein 4 (BRD4) in TNBC cell lines regulates SNAI1 in a PKD1-dependent manner, resulting in decreased migration and invasion in breast cancer cells and reduced tumor growth and metastasis in xenograft models (Lu et al., 2020). On the other hand, treatment of PKD1 negative, PKD2/PKD3-positive TNBC MDA-MB-231 cells with the pan-PKD inhibitor CRT0066101 decreased SNAI1 levels as well as other EMT markers (Durand et al., 2016) suggesting that PKD2 and PKD3, possibly acting as heterodimers, have opposite functions to PKD1 with respect to SNAI1 regulation. At first glance, this seems contradictory because it is likely, although not proven, that PKD1, PKD2, and PKD3 phosphorylate the same serine position in SNAI1, which would create a phosphodegron in one case while stabilizing the protein in the other. However, PKD2/3-mediated SNAI1 phosphorylation has not been demonstrated in this context, and the decrease in SNAI1 levels on pharmacological inhibition of PKD2 and PKD3 may therefore be an indirect effect. Notably, secreted matrix metalloproteinases (MMPs), especially MMP-3, can induce sustained EMT in a SNAI1-dependent manner in mouse mammary epithelial cell lines (Lochter et al., 1997; Przybylo and Radisky, 2007). Because PKD2 activity regulates the secretion of MMPs in other tumor cell lines (Eiseler et al., 2016), it could be speculated that pharmacological inhibition of PKD2/PKD3 activity in MDA-MB-231 cells blocks MMP secretion, thereby attenuating their autocrine signaling that maintains the cells in a post-EMT state.

In summary, loss of PKD1 has been widely accepted as a marker of aggressiveness in various cancer types and model systems, including its correlation with poorer prognosis in breast cancer patients. However, as previously discussed by Eiseler and colleagues (Eiseler et al., 2012), this does not change the fact that PKD1 may well play a significant role in tumor growth, possibly during tumor formation, either in the early stages of disease or after metastasis. Its silencing, on the other hand, would be beneficial at a later stage in promoting invasiveness and metastasis, as the cells then adopt a more motile phenotype. Indeed, Karam et al. showed that overexpression of PKD1 in noninvasive MCF-7 cells (which do not naturally express PKD3 (Huck et al., 2013) has tumor-promoting functions *in vitro* and *in vivo* (Karam et al., 2012). Another important factor in the regulation of EMT is the transcription factor Twist1, and in a recent study published by Georgess and colleagues, RNAseq profiling of Twist1-inducible breast organoids during epithelial dissemination showed upregulation of PKD1 and none of the other PKD family members (Georgess et al., 2020). In principle, these data contradict previous findings suggesting PKD1 as an EMT repressor in breast cancer [reviewed in detail in (Durand et al., 2016)]. However, another study has shown that Twist1 suppresses ER expression in breast cancer cells (Fu et al., 2012). Given that loss of ER promotes PKD3 expression (Borges et al., 2015) and that the inhibitors used block all PKDs, it would be interesting to control PKD3 protein levels in this particular situation to rule out an effect of PKD3 in Twist1-driven dissemination, although *PRKD3* transcript levels were not significantly upregulated in Twist1 organoids. This opens the possibility for new studies addressing whether PKD1 re-expression actually occurs *in vivo*, what its effects and regulatory mechanisms are, and whether it co-exists with other PKD isoforms.

In light of the above, can PKD-regulated membrane trafficking drive EMT in breast cancer? On the one hand, direct phosphorylation of SNAI1 or E-cadherin by PKD1 would not necessarily imply altered membrane trafficking. Rather, it would explain other studies showing that PKD1 negatively controls invasion by downregulating MMP9 expression in breast cancer cells (Qin et al., 2015). On the other hand, upon EMT, MMPs and other ECM-related molecules are frequently secreted to degrade and alter the structure of the ECM, which promotes invasion (Hielscher et al., 2016; Kai et al., 2019) or contributes to chemotherapy resistance in TNBC (Saatci et al., 2020). However, since PKD1 expression is lost during the transition to the invasive phenotype, PKD2 and/or PKD3 would need to control MMP secretion. Indeed, Eiseler and colleagues described the PKD2-dependent assembly of a multiprotein complex at the TGN, which promotes the secretion of MMP2- and 7 in mouse embryonic fibroblasts (Eiseler et al., 2009a). Moreover, knockdown of PKD3 in prostate cancer cells inhibited the secretion of tumor-promoting factors amongst them MMP-9, IL-6, Il-8 and GROα (Lavalle et al., 2012). Whether this is also the case in TNBC cell lines and contributes to the PKD2/3-dependent tumor-promoting phenotype awaits investigation.

### 3.3 Oncogenic role of PKD3 in breast cancer

In addition to loss of PKD1 expression, breast cancer cells undergo an isoform switch towards PKD3, as previously mentioned, which has been commonly linked to tumor progression [initially reported in (Borges et al., 2015)]. In this study, Borges et al. established the correlation between PKD3 and ERα expression in breast cancer, as ERα binds to the PRKD3 promoter, inhibiting PKD3 expression. Because of the altered expression of the hormone receptors (including loss of ER) that characterizes invasive breast cancer, PKD3 becomes highly upregulated in ER− breast cancer. Nevertheless, whether this is the only mechanism regulating PKD3 expression in breast or other cancer types remains still unclear.

We now recapitulate recent studies on how PKD3 expression regulates tumor-supporting functions, such as proliferation, migration, invasion, EMT and stemness. For instance, in a TNBC cell line, HCC 1806, PKD2 and specially PKD3 were found to be key regulators of cell proliferation and tumor growth. Furthermore, PKD inhibitors reduced cell growth in cells expressing higher levels of PKD3 and outperformed inhibition of PKC, suggesting that PKD3 is the main isoform driving tumor progression in breast cancer (Hao et al., 2013). Furthermore, mTORC1-S6K signalling pathway, an important driver of cell proliferation, was found downstream PKD3 in TNBC cell lines; PKD3 depletion led to impaired activation of mTORC1 at endolysosomal membranes, showing for the first time that PKD3 is necessary to maintain the integrity of the endolysosomal system in TNBC cell lines (Huck et al., 2013). In NSCLC cells, however, depletion of PKD1 enhanced the activation of S6K in response to phorbol ester, while the constitutive active kinase impaired S6K phosphorylation (Ni et al., 2015). Even though it is not known whether this mechanism also occurs in breast cancer cells, both observations together could indicate that PKD1 silencing and PKD3 overexpression, through different mechanisms, mutually converge in the activation of the mTORC1-S6K axis. To note, in this first study (Huck et al., 2013), neither Akt nor Erk1/Erk2 phosphorylation were modulated by PKD3 overexpression, depletion or pharmacological inhibition. However, a recent study found PKD3 to have a role in proliferation of MDA-MB-231 and MDA-MB-468 cells by activating the Erk1/c-Myc pathway (Liu et al., 2020). In conclusion, the specific mechanism regulating PKD3-dependent activation of mTORC1 in TNBC cells needs to be further studied, but these results open the door to uncover new tumor-promoting functions of PKD3 at different sub-cellular localizations, such as the endolysosomal system. In a recent study, PKD3 was found to inhibit lysosomal-dependent degradation of the stress-activated chaperone clusterin, thereby promoting clusterin secretion and TNBC tumor growth in a xenograft mouse model (Liu et al., 2021). Interestingly, depletion of PKD3 led to accumulation of clusterin in Lamp1-positive vesicles. Clusterin is a secretory protein whose biosynthesis occurs via the conventional pathway of exocytosis. Considering the function of PKD2/PKD3 dimers in the biogenesis of exocytic transport vesicles at the TGN (Malhotra & Campelo, 2011), it is conceivable that loss of PKD3 at this compartment causes the mis-sorting of secretory cargo proteins to the endolysosomal system. In line with that, secreted clusterin levels dropped upon treatment of human TNBC organoids and TNBC xenograft mouse with the pan-PKD inhibitor CRT006101 (Liu et al., 2021).

Another study showed endogenous PKD3 localization to VAMP2-positive vesicular structures in the cytosol, apart from the nucleus, in TNBC HCC1806 cells, that partially colocalized with the endocytic compartment (Lu et al., 2007). However, when considering these results, it should be noted that the endogenous kinase was detected with an affinity-purified polyclonal antibody, the specificity of which remains to be validated in immunofluorescence (Lu et al., 2007). In this context, PKD1 has been implicated in αvβ3 and α5β1 integrin and EGFR recycling via phosphorylation of rabaptin-5, an effector of Rab5 found in endosomal membranes. By promoting αvβ3 recycling, rabaptin-5 phosphorylation negatively affects invasion in fibronectin-rich matrices, whereas in fibronectin-deficient microenvironments, where invasion depends on αvβ3 rather than α5β1, rabaptin-5 phosphorylation is a strong driver of tumor cell invasion (Christoforides et al., 2012). Although it is not yet clear whether this specific phosphorylation occurs at an endogenous level in TNBC cells, these studies demonstrate an involvement of PKD in the endocytic pathway, which may have implications for breast cancer progression. In breast cancer, high-level fibronectin expression levels are linked with decreased patient survival (Shinde et al., 2018). The fact that rabaptin-5 phosphorylation negatively affects invasion in this context and that loss of PKD1 promotes invasion while loss of PKD3 blocks invasion supports the idea that PKD1/PKD2 and PKD2/3 heterodimers have different substrate portfolios in breast cancer cells, possibly based on their different localization, and thus have opposing functions.

In another line, Lieb and colleagues demonstrated for the first time the importance of PKD3 for the CSC population in TNBC (Lieb et al., 2020). CSC are a subpopulation of tumor cells that are associated with tumor initiation, resistance to chemotherapeutics, tumor relapse and metastases (Zheng et al., 2021). In this study, GEF-H1-dependent PKD3 activity was required for stem cell maintenance in TNBC cells, as evidenced by decreased oncosphere formation, expression of stem cell markers, or ALDH1A1 activity upon PKD3 loss or pharmacological inhibition compared with control. In contrast, these differences were not observed in non-invasive MCF-7 cells expressing only minimal levels of PKD3 (Lieb et al., 2020). Whether this effect is due to autocrine or paracrine signaling requires further investigation, but given the importance of the GEF-H1/RhoA/PKD axis in exocytosis in epithelial cells (Eisler et al., 2018), this hypothesis is plausible. For example, microRNAs (miRNAs) commonly found in exosomes play a central role in regulating CSC properties by targeting specific signaling pathways that generate, maintain, and propagate CSC-like phenotypes, such as Wnt/β-catenin, JAK/STAT, or Notch [reviewed in (Humphries et al., 2021)]. Indeed, miR-34a targeting and silencing of PKD1, as described in drug-resistant MCF-7-ADR cells, contributed to drug resistance by promoting stem cell formation through alteration of the GSK3/β-catenin signaling pathway (Kim et al., 2016).

### 3.4 Do PKD2 and PKD3 have redundant functions in breast cancer?

To date, several substrates have been found to be phosphorylated by members of the PKD family. However, whether these phosphorylations are mediated by specific PKD isoforms is largely unclear, as often only one isoform has been studied and the kinases phosphorylate the same consensus sequence *in vitro* (Hutti et al., 2004; Döppler et al., 2005). Nevertheless, some of these studies include silencing of all PKD isoforms to determine substrate-specificity. For instance, in HEK293T cells, PI4KIIIβ was phosphorylated only by PKD1 and PKD2 but not by PKD3 at the TGN (Hausser et al., 2005). Furthermore, GIT1 was described as a specific substrate for PKD3 in TNBC cells, and PKD3-dependent phosphorylation modulated its localization in motile cytoplasmic complexes (Huck et al., 2012). Of note, loss of GIT1 in ER− breast cancer tumors increased Notch signaling and the cancer stem cell pool (Zhang et al., 2022), reflecting the phenotype of increased PKD3 expression (Lieb et al., 2020). Cytosolic GIT1 thereby directly interacts with the intracellular domain of Notch (ICD) and blocks its transport from the cytoplasm to the nucleus (Zhang et al., 2022). Considering that the localization of GIT1 in cytoplasmic complexes occurs through PKD3-mediated phosphorylation, this may represent a sequestration mechanism that serves to prevent the interaction between GIT1 and Notch, thereby allowing Notch signaling and maintenance of the stem cell pool. These results suggest not only that PKD3 has specific targets in TNBC, but also that this substrate specificity could be explained by differential localization of this isoform. Moreover, unlike PKD3, PKD2 was activated only in response to RhoA activation in MDA-MB-468 cells, but not under basal conditions (Döppler et al., 2014). Similarly, only PKD3, but not PKD2, was activated in response to GEF-H1 in TNBC cells (Lieb et al., 2020); and silencing of PKD2 in PKD2/PKD3-positive MDA-MB-231 cells was not sufficient to alter cell migration (Peterburs et al., 2009), because basal PKD3 activity contributes to directional cell migration by regulating cofilin (Döppler et al., 2014). These disparate observations lead us to speculate whether in TNBC PKD2 and PKD3 are indeed interdependent for dimer formation and activity, as previously shown in HeLa cells (Bossard et al., 2007), or whether, instead, abnormal expression of PKD3 alone is sufficient to increase its activity through the formation of homodimers, thereby promoting tumor progression. In this context, the higher PKD3 levels during breast cancer progression compared with PKD2 and the near absence of PKD1 expression suggest that PKD3 activity may be independent of the expression of the other isoforms, which would support the idea of an autonomously functioning oncogene. In this context, it is particularly important to identify endogenous PKD3 localization in TNBC cells, for which new tools such as CRISPR/Cas9-mediated gene editing could be useful.

**FIGURE 4:**
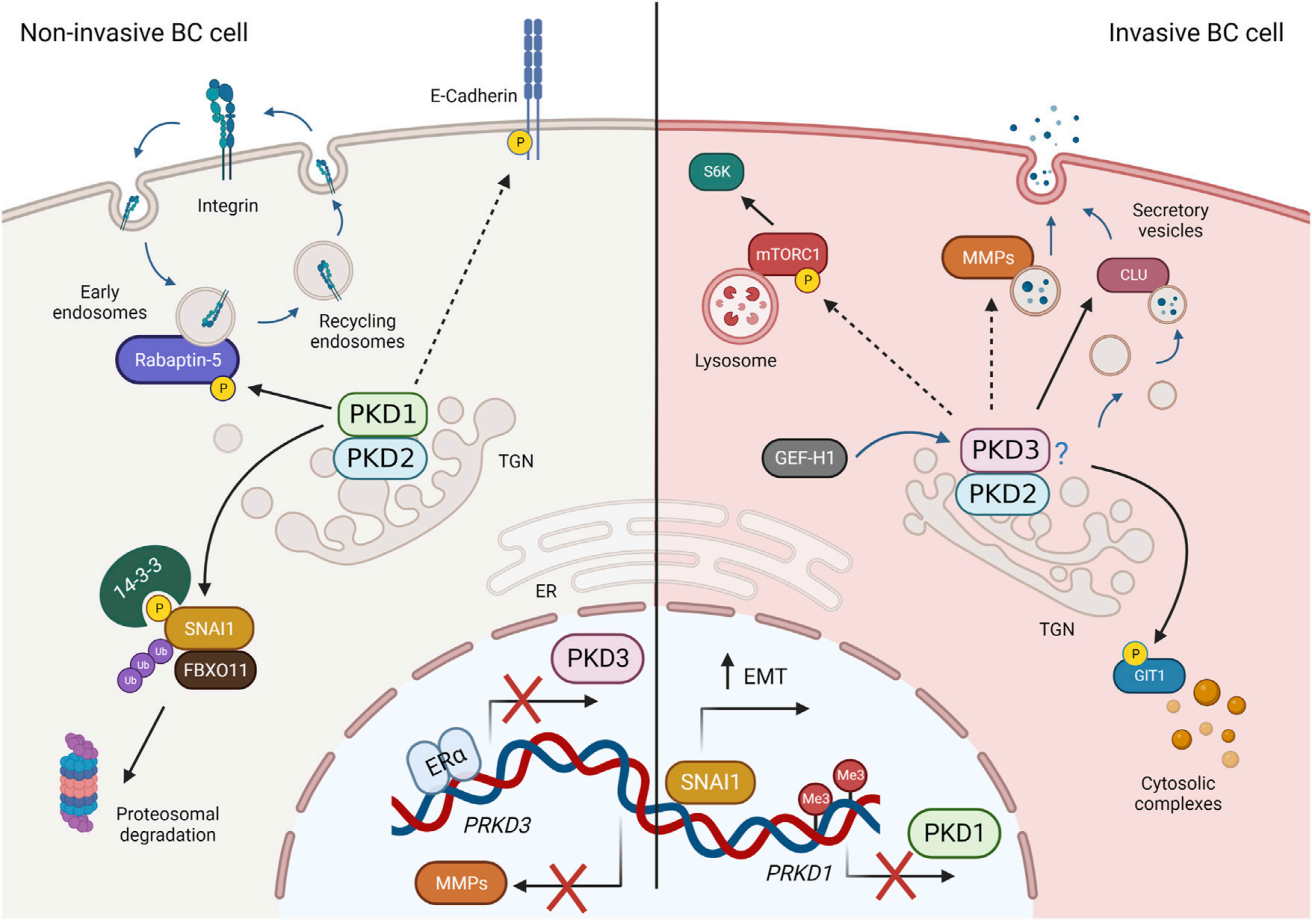
PKD-regulated membrane traffic and signaling pathways in breast cancer (BC). Left: In non-invasive breast cancer cells, PKD3 expression is repressed by ERα. PKD1 and PKD2 (presumably as heterodimers) phosphorylate SNAI1, leading to its degradation, promote avß3 integrin recycling through Rabaptin-5, stabilize cell-cell contacts via E-cadherin, and block expression of MMPs, thereby negatively regulating invasion, migration, and EMT. Right: In invasive BC cells (basal-like, TNBC), PKD1 expression is suppressed, whereas PKD3 (and PKD2) expression is increased. PKD2 and PKD3, which presumably act as heterodimers, are activated downstream of GEF-H1 and control MMP and clusterin (CLU) secretion, mTORC1 activity, and GIT1 localization to promote proliferation, stem cells, invasion, and EMT. Dashed arrows represent indirect interactions or processes shown in other cell types. For more details, see text. Created in BioRender.

PKD activation, as mentioned earlier, requires binding to DAG in membranes. Therefore, it may also be beneficial to investigate how DAG distribution is regulated in breast cancer cells. However, this requires cell culture models that are more complex, and in which lipid composition changes dynamically between different cells in the TME such as CAFs, immune cells and endothelial cells, consistent with physiological conditions. For example, a recent work examined the lipid profile of 2D monolayer and 3D multicellular tumor spheroids from colon carcinoma cells and found significant differences, not only when comparing the different culture systems, but also in different regions of the tumor spheroid (Tobias and Hummon, 2022). Another example is the use of co-culture systems in which cells exchange lipids and other signaling molecules via EVs. Increased unconventional secretion in cancer cells releases EVs containing pro-oncogenic molecules that alter the TME and thus influence tumor growth and metastasis through paracrine signaling (Green et al., 2015; Becker et al., 2016; Ciardiello et al., 2016). A recent lipidomic analysis of cells and EVs derived from high- and low-metastatic TNBC cell lines showed increased enrichment of unsaturated DAGs in EVs derived from high-metastatic cells compared with low-metastatic cells, whereas only a slight increase in DAG was seen in the cells themselves. Moreover, these DAG-enriched EVs were able to activate endogenous PKD in endothelial cells (Nishida-Aoki et al., 2020). Strikingly, in pancreatic cancer cells, loss of PKD1 enhanced secretion of EVs that promoted metastasis of xenograft and pancreatic tumors to lung in mice (Armacki et al., 2020), demonstrating a role for PKD in aberrant EV secretion. To evaluate the effects of PKD-dependent secretion in breast cancer cells, new studies could analyze the soluble factors and EVs present in the supernatant, as well as the deposited ECM, and how they affect the TME. In this context, the contribution of PKD isoforms by modulating their expression levels could then be further elucidated. In favor of the hypothesis that PKD controls the cancer secretome is a recent study in prostate cancer cells. Here, PKD3 is required for the secretion of tumor promoting factors such as MMP9, IL-6 and IL-8 (Lavalle et al., 2012). However, because loss of PKD3 also greatly reduced cell proliferation *in vitro* and tumor growth *in vivo* (Lavalle et al., 2012), and secretion and cell growth are closely linked, it is not clear whether the decreased cell growth is the consequence or cause of the diminished secretion. A summary of membrane traffic and signaling pathways regulated by PKD isoforms in breast cancer is presented in Figure 4.

## 4 Conclusion and outlook

Our knowledge of the role of the PKD family in cancer progression has increased greatly in recent years. In particular, in breast cancer, the loss of PKD1 and increase in PKD3 expression in invasive ductal carcinomas underscores the existence of isoform-specific functions. This argues for isoform-specific inhibition of PKD activity to block tumor-promoting function. To date, however, only pan-PKD inhibitors exist, and although they have shown therapeutic potential in various cancer models (Harikumar et al., 2010; Ochi et al., 2011; Borges et al., 2015; Lieb et al., 2020), small-molecule PKD inhibitors have not yet entered clinical trials. This may be because PKD isoforms play an essential role in development and tissue and organ function (Matthews et al., 2010; Löffler et al., 2018; Zhang et al., 2020) and thus side effects might be expected. In addition, pan-PKD inhibitors have off-target effects to some extent, as the PKD inhibitor CRT0066101 has shown activity against many other protein kinases (Guedán et al., 2017). The development of isoform-specific small molecule PKD inhibitors is urgently needed, but this seems to be a challenge due to the highly conserved kinase domains. Alternatively, compounds that are synergistic with PKD inhibition would allow the use of low doses with minimal toxicity. An example of this was the combination of the CRT006101 with the chemotherapeutic agent paclitaxel to reduce tumor growth *in vitro* and *in vivo* in TNBC cells (Lieb et al., 2020). On the other hand, re-expression of PKD1 to rescue its tumor suppressive functions in terms of invasiveness and metastasis by general suppression of methylation using the epi-drug decitabine has shown promising results in *in vivo* models (Borges et al., 2013), but the compound is not specific to PKD1. Therefore, new methods need to be developed to exclusively modulate the desired isoform. Here, the application of next-generation techniques such as auxin-inducible degradation technology or proteolysis-targeting chimeras (PROTACs) enables rapid and controlled depletion of proteins to detect primary molecular responses and avoid secondary, indirect effects of protein dysregulation (Beyer et al., 2015; Schapira et al., 2019). In addition, epigenetic editing could be used as a promising approach to reactivate PKD1 expression by targeted demethylation in a controlled manner (Falahi et al., 2014).
